# The impact of CFNS-causing *EFNB1 *mutations on ephrin-B1 function

**DOI:** 10.1186/1471-2350-11-98

**Published:** 2010-06-17

**Authors:** Roman Makarov, Bernhard Steiner, Zoran Gucev, Velibor Tasic, Peter Wieacker, Ilse Wieland

**Affiliations:** 1 Institut für Humangenetik, Universitätsklinikum, Otto-von-Guericke-Universität, Magdeburg, Germany; 2 Institut für Medizinische Genetik, Universität Zürich, Schwerzenbach, Switzerland; 3 Department of Pediatric Endocrinology and Genetics, University Children's Hospital, Skopje, Former Yugoslav Republic of Macedonia; 4 Institut für Humangenetik, Universitätsklinikum, Westfälische Wilhelms-Universität, Münster, Germany

## Abstract

**Background:**

Mutations of *EFNB1 *cause the X-linked malformation syndrome craniofrontonasal syndrome (CFNS). CFNS is characterized by an unusual phenotypic pattern of inheritance, because it affects heterozygous females more severely than hemizygous males. This sex-dependent inheritance has been explained by random X-inactivation in heterozygous females and the consequences of cellular interference of wild type and mutant *EFNB1*-expressing cell populations. *EFNB1 *encodes the transmembrane protein ephrin-B1, that forms bi-directional signalling complexes with Eph receptor tyrosine kinases expressed on complementary cells. Here, we studied the effects of patient-derived *EFNB1 *mutations predicted to give rise to truncated ephrin-B1 protein or to disturb Eph/ephrin-B1 reverse ephrin-B1 signalling. Five mutations are investigated in this work: nonsense mutation c.196C > T/p.R66X, frameshift mutation c.614_615delCT, splice-site mutation c.406 + 2T > C and two missense mutations p.P54L and p.T111I. Both missense mutations are located in the extracellular ephrin domain involved in Eph-ephrin-B1 recognition and higher order complex formation.

**Methods:**

Nonsense mutation c.196C > T/p.R66X, frameshift mutation c.614_615delCT and splice-site mutation c.406+2T > C were detected in the primary patient fibroblasts by direct sequencing of the DNA and were further analysed by RT-PCR and Western blot analyses.

The impact of missense mutations p.P54L and p.T111I on cell behaviour and reverse ephrin-B1 cell signalling was analysed in a cell culture model using NIH 3T3 fibroblasts. These cells were transfected with the constructs generated by *in vitro *site-directed mutagenesis. Investigation of missense mutations was performed using the Western blot analysis and time-lapse microscopy.

**Results and Discussion:**

Nonsense mutation c.196C > T/p.R66X and frameshift mutation c.614_615delCT escape nonsense-mediated RNA decay (NMD), splice-site mutation c.406+2T > C results in either retention of intron 2 or activation of a cryptic splice site in exon 2. However, c.614_615delCT and c.406+2T > C mutations were found to be not compatible with production of a soluble ephrin-B1 protein. Protein expression of the p.R66X mutation was predicted unlikely but has not been investigated.

Ectopic expression of p.P54L ephrin-B1 resists Eph-receptor mediated cell cluster formation in tissue culture and intracellular ephrin-B1 Tyr324 and Tyr329 phosphorylation. Cells expressing p.T111I protein show similar responses as wild type expressing cells, however, phosphorylation of Tyr324 and Tyr329 is reduced.

**Conclusions:**

Pathogenic mechanisms in CFNS manifestation include impaired ephrin-B1 signalling combined with cellular interference.

## Background

Mutations in *EFNB1 *(OMIM 300035 [1]), encoding the transmembrane protein ephrin-B1, have been detected in the majority of patients with familial and sporadic craniofrontonasal syndrome [2,3]. Craniofrontonasal syndrome (CFNS; OMIM 304110 [1]) is an X-linked developmental malformation syndrome with variable phenotypic expression. It affects females more severely than males which is quite unusual for X-linked genetic diseases [4]. The unusual phenotypic pattern of inheritance has been explained by heterozygosity for an *EFNB1 *mutation and the consequences of random X-inactivation in the female patients [2].

Ephrin-B1 forms signalling complexes with Eph receptor tyrosine kinases that are involved in cell sorting, migration and adhesion, midline fusion, axon guidance, neural plasticity and synaptogenesis [5,6]. In many embryonic and adult tissues, ephrin ligand and Eph receptor show complementary expression that function in bi-directional cell signalling [7,8]. Contact of Eph-receptor expressing cells with ephrin-B1-expressing cells drives forward signalling in the Eph-expressing cells and reverse signalling in the ephrin-B1-expressing cells. Forward signalling leads to cell repulsion, whereas reverse signalling appears to affect mostly cell-cell communication through gap junctions [6,9]. Upon Eph/ephrin binding of opposing cells, several tyrosines (corresponding to human Tyr313, Tyr317, Tyr324, Tyr329, Tyr343 and Tyr344) in the ephrin cytoplasmic tail are phosphorylated by Src family tyrosine kinases (SFKs), that co-localize in lipid rafts and use phosphotyrosine-independent docking mechanisms [10,11,8]. Phosphorylated ephrin-B1 serves as a docking site for SH2-containing adaptor proteins, such as Grb4, which then activate signalling pathways ultimately leading to changes in actin cytoskeleton and focal adhesion [12,13]. In addition, other signalling molecules are recruited such as the GTP exchange factor PDZ-RGS3 and the signal transducer and activator of transcription 3 (STAT3) by highly conserved C-terminal motifs [14,15]. Phosphorylation of Tyr324 and Tyr329 was shown to be most important for ephrin-B1 reverse signalling [16-18]. Bi-directional signalling leads to restriction of cell intermingling and communication, particularly, at cellular interfaces and tissue boundaries [19]. In the pathological condition existing in CFNS female patients, mutant and wild type cellular compartments have been proposed to cause cellular interference that leads to disturbed border formation [2,3].

*EFNB1 *gene consists of 5 exons. The extracellular ephrin domain is encoded by exons 2 and 3, the transmembrane and intracellular domains are encoded by exon 5. The major types of *EFNB1 *mutations (up to 55%) are frameshift, nonsense, and splice site mutations that lead to premature termination codons (PTCs). Missense mutations constitute about 42% of all *EFNB1 *mutations [3,20]. Most of them occur in exons 2 and 3, leading to the exchange of amino acid residues that are important for receptor-ligand interaction and signalling. Loss of gene function has been proposed for most mutations and has been shown for some of them, but it has not been proven for missense mutations, splice site mutations, and for mutations causing premature termination in exons 4 and 5 [21,2,25].

The concept of cellular interference appears to be not unique to CFNS. Dibbens et al. [26] described the molecular cause of epilepsy and mental retardation limited to females (EFMR, OMIM 300088 [1]). This X-linked disorder affects females, while male carriers are unaffected. EFMR is caused by mutations in *PCDH19 *gene encoding the cell-cell adhesion molecule protocadherin 19. Like in CFNS, somatic mosaicism may cause cellular interference leading to malformations in the brain and development of epilepsy [27]. This pathogenic mechanism has been strongly supported by a mosaic male patient harbouring a *PCDH19 *mutation, who was identified in a cohort of patients with Dravet syndrome-like epileptic encephalopathy [28].

Here we analysed the impact of patient-derived *EFNB1 *on ephrin-B1 reverse signalling *in vitro *and in a cell culture model.

## Methods

### Cell culture

Genetic testing of the patients was performed after written informed consent from the patients' parents and complies with the tenets of the declaration of Helsinki. Biopsies of CFNS patients were obtained from skin (c.196C > T/p.R66X and c.406+2T > C) or following surgical therapeutic interventions (c.614_615delCT). Patient fibroblast cultures were established and maintained according to standard cell culture conditions and harvested for genomic DNA and total RNA or protein isolation.

NIH 3T3 cells were cultivated in tissue culture flasks (Cellstar^®^) with Dulbecco's modified Eagle's medium (DMEM, Sigma) containing 15% fetal calf serum (FCS, Sigma) in a 5% CO_2 _atmosphere at 37°C.

### Mutation detection

Genomic DNA and RNA from cultured cells were isolated using standard protocols. Mutations were detected by direct sequencing with the DYEnamic ET terminator cycle sequencing kit (GE Healthcare Europe), and run on a MegaBace sequence analyser (GE Healthcare). Sequences were processed by DNASIS software (MiraiBio, Alameda, USA). The sequencing data were compared with *EFNB1 *reference sequence GenBank accession number NM_004429.4 and NG_008887.71 [29] and Ensembl number ENST00000204961 [30]. Mutations were confirmed by exon-specific PCR amplification and restriction enzyme digestion in all of the primary cell cultures. PCR primers were 5'-CAAGTTCCTGAGTGGGAAGG-3' and 5'-GTGTGGCCATCTTGACAGTG-3' producing a 455 bp product from exons 2-4 for analysing c.196C > T/p.R66X. Primer pair 5'-GGCTCTTGTCCGCTTCCCTG-3' and 5'-CCAGTCTTCAAAGGGGATCA-3' producing a 502 bp fragment containing exon 2 was used for analysing c.406+2T > C, and primer pair 5'-AGGAACAGTCAGCCAGGGG-3' and 5'-GGGGAGCAGGCGTAGGGTTA-3' producing a 377 bp product containing exon 4 was used for analysing c.614_615delCT. Primers were designed using the program Primer3 v.0.4.0 [31]. The PCR products were cleaved with restriction enzymes: *Ava*I detecting c.196C > T/p.R66X, *Hinf*I detecting c.614_615delCT and *BfuA*I detecting c.406+2T > C (all enzymes were from New England Biolabs).

### RT-PCR and cloning

For expression analysis, total RNA was reverse transcribed using SuperScript™ One-Step™ RT-PCR System (Life Technologies) as recommended by the supplier. Primer pair 5'-CAAGTTCCTGAGTGGGAAGG-3' and 5'-GTGTGGCCATCTTGACAGTG-3' was used to amplify a 455 bp product from exons 2-4, and primer pair 5'-ATCATGAAGGTTGGGCAAGA-3' and 5'-TGGGGGCAGTAGTTGTTCTC-3' was used to amplify a 467 bp product from exons 4 and 5 of *EFNB1*. RT-PCR products from cells carrying nonsense mutation c.196C > T/p.R66X and frameshift mutation c.614_615delCT were cleaved with *Ava*I and *Hinf*I, respectively. RT-PCR products obtained from the c.406+2T > C *EFNB1 *allele were cloned into pGEM-Teasy vector (Promega) and sequenced as described [20].

### Generation of the mutant *EFNB1 *cDNA constructs by site-directed *in vitro *mutagenesis

*EFNB1 *RNA was prepared from placenta and reverse transcribed as described above. Amplification of *EFNB1 *cDNA was performed using primer pair 5'-GGCAGAGGAAGGCGAGGCGA-3' and 5'-GCAAGGGGAGGGGGTGTG-3' that generates an 1.2 Kb product. This RT-PCR product was cloned into the pCR 2.1 vector (Invitrogen). Mutant *EFNB1 *cDNA containing c.161C > T/p.P54L and c.332C > T/p.T111I mutations were generated using QuikChange^® ^II Site-Directed Mutagenesis Kit (Stratagene) according to the kit's protocol and *EFNB1*-specific primers: 5'-GGGCTTGGTGATCTATCTGAAAATTGGAGACAAGC-3' and 5'-GCTTGTCTCCAATTTTCAGATAGATCACCAAGCCC-3' for c.161C > T, 5'-CAGAGCAGGAAATACGCTTTATAATCAAGTTCCAGGAGTTCA-3' and 5'-CTGAACTCCTGGAACTTGATTATAAAGCGTATTTCCTGCTCTG-3' for c.332C > T with nucleotide exchanges underlined. Primers were designed using the web-based primer software program (Stratagene) [32]. In the patients, nucleotide exchange c.332C > T leads to the codon exchange ACC > ATC and amino acid exchange threonine to isoleucine. In this work nucleotide exchange c.332_333CC > TA was used. It leads to the codon exchange ACC > ATA and the same amino acid exchange. This was done because the threonine codon ATA is more frequently used then ATC.

The presence of mutations was confirmed by sequencing with the AutoRead™ Sequencing Kit (Amersham Biosciences) according to the kit's protocol. Wild type and mutant *EFNB1 *cDNA inserts were recloned in pcDNA 3.1(+) vector (BD Biosciences) using *EcoRI *restriction (MBI Fermentas).

### NIH 3T3 transfection

NIH 3T3 cells were placed on a 6-well plate (1 × 10^5 ^cells/well, Greiner Labortechnik). Cells were cultivated until they reached 70% confluence (usually within 24 h). Plated cells were co-transfected with pcDNA 3.1(+) vector containing the mutant or the wild type *EFNB1 *cDNA (4 μg/well) and the pEGFP-N3 vector (4 μg/well, BD Biosciences). Transfections were done using PerFectin™ Transfection reagent (PeqLab) according to the supplier's recommendations. Transfection efficiency was measured using fluorescent cytometry cell sorting (FACS) 24 h post transfection as described below.

### FACS analysis

Transfected cells were washed in PBS (Sigma) and treated with trypsine (Sigma) 24 h post-transfection. After the treatment, PBS was added and part of the cells was taken for the FACS analysis using the ectopic GFP-fluorescence and BD FACSCanto™ Flow Cytometer (BD Bioscience), 3 × 10^4 ^events were counted. Untransfected NIH 3T3 cells served as controls.

### NIH 3T3 stimulation

Stimulation of NIH3T3 cells with EphB2-receptor was performed according to a modified protocol of Davy et al. [9]. Briefly, NIH 3T3 cells were transfected and cultivated for 32 h. Cells were prepared for EphB2-Fc (R&D Systems) stimulation by washing in PBS and incubation in DMEM containing 0.5% FCS for 16 h. EphB2-Fc/Fc (50 μg/ml) were pre-clustered with anti-human rabbit IgG (100 μg/ml, R&D Systems) in DMEM for 30 min at room temperature. Starvation medium was aspirated, pre-clustered EphB2-Fc/Fc containing medium was diluted to the final EphB2-Fc/Fc concentration of 4 μg/ml and added to the cells. After time intervals of 5 to 30 min of stimulation, cells were washed in PBS and cultivated in DMEM with 15% FCS as described above. As a control, stimulation with Fc (R&D Systems) was performed. Analysis of cluster formation was done 24 h after stimulation. Pictures were taken by fluorescent microscopy method (Axiovert 25 Inverse Microscope and AxioCam MRc5 0450-354, Carl Zeiss).

### Western blot analysis

Patient fibroblasts were lysed with RIPA buffer [33] and used for the SDS-PAGE and Western blot analysis using anti-ephrin-B1 antibody (A-20, Santa Cruz Biotechnology, Inc.). Immediately after the EphB2-Fc stimulation of NIH 3T3, cells were washed in PBS and lysed in RIPA buffer containing PhosphoStop solution (Roche). Lysates were used for the Western blot analysis using Phospho-Ephrin B (Tyr324/329) antibody (Cell Signaling Technology^®^) and anti-ephrin-B1 antibody (A-20). According to the manufacturer (Santa Cruz), rabbit polyclonal antibody A-20 was raised against a 20 amino acid peptide encoded by exon 3 that maps in the extracellular ephrin-B1 domain.

All the experiments were performed at least twice.

## Results

**Figure 1:**
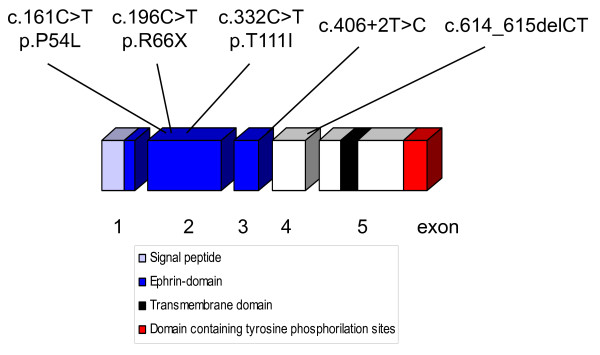
**Schematic representation of the *EFNB1 *coding cDNA**. The boxes represent the 5 exons with the functional domains of the protein shown in different colours. The position and type of the investigated mutations is shown in the upper part.

Expression of *EFNB1 *nonsense mutation c.196C > T/p.R66X that is located in exon 2, frameshift mutation c.614_615delCT in exon 4 and splice-site mutation c.406+2T > C at the junction of exons 2 and 3 was analysed, respectively. Two missense mutations located in exon 2 of *EFNB1 *were functionally studied (Figure 1). Both, missense mutation p.P54L and p.T111I, likely change the conformation of the extracellular globular part of the ephrin-B1 protein that interacts with the Eph-receptor [2,34]. Their impact on ephrin-B1 reverse signalling was investigated in a cell culture model. All mutations investigated were found in CFNS patients.

### Molecular analysis of protein-truncating *EFNB1 *mutations in patient fibroblasts

**Figure 2:**
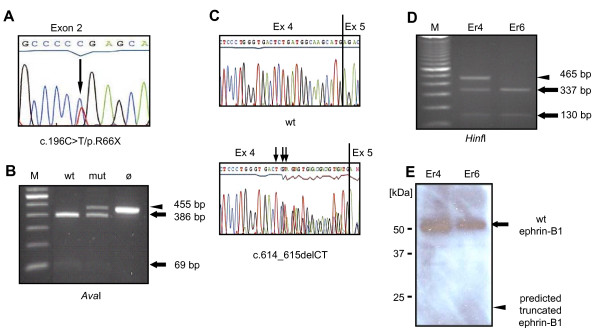
**Expression of *EFNB1 *transcript and protein in primary patient fibroblasts harbouring heterozygous nonsense mutation c.196C > T/p.R66X and heterozygous frameshift mutation c.614_615delCT**. (A) The mutation c.196C > T/p.R66X has been shown by direct sequencing of the cDNA. Nucleotide exchange C > T (indicated by an arrow) creates a premature termination codon TGA in exon 2. (B) Wild type and mutant *EFNB1 *RNA were expressed in a patient fibroblast culture (lane mut). In the control (lane wt) only wild type *EFNB1 *RNA is expressed. Wild type and mutant transcripts were distinguished by RT-PCR followed by cleavage with restriction enzyme *Ava*I. Wild type allele is indicated by arrows, mutant allele is indicated by an arrowhead. In lane Ø the RT-PCR product prior to cleavage is shown. (C) Direct sequencing of *EFNB1 *cDNA of a control (upper panel) and mutation c.614_615delCT (lower panel). Deletion of the CT dinucleotide creates a premature termination codon TGA in exon 4 (indicated by arrows). (D) Wild type and mutant *EFNB1 *RNA were expressed in a patient fibroblast culture (lane Er4). In the control (lane Er6) only wild type *EFNB1 *RNA is expressed. Wild type and mutant transcripts were determined by RT-PCR followed by cleavage with restriction enzyme *Hinf*I. Wild type allele is indicated by arrows, mutant allele is indicated by an arrowhead. Size markers are shown in lane M (100 bp DNA ladder, Invitrogen). (E) Western blot analysis of ephrin-B1 expression in lysates of patient fibroblast cultures. Er4 and Er6 show an approximately 50 kDa protein (indicated by an arrow). No smaller truncated protein corresponding to c.614_615delCT was detected at the predicted molecular weight of ≈20 kDa in Er4 (expected size indicated by an arrowhead) using an anti-ephrin-B1 antibody. Protein sizes were determined using Precision Plus Protein™ Standards Dual Color (BIO-RAD).

**Figure 3:**
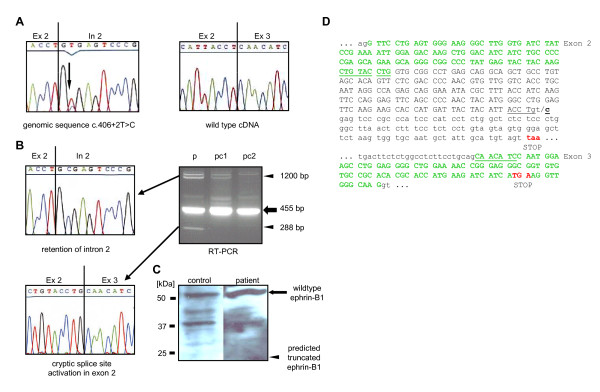
**Sequence of *EFNB1 *splice site mutation c.406+2T > C and expression of *EFNB1 *transcript and protein in primary patient fibroblasts**. (A) The mutation has been detected by direct sequencing of genomic DNA. Nucleotide exchange T > C in intron 2 at the splice donor site is indicated by an arrow. The major transcript expressed in patient fibroblasts was the wild type allele. (B) Wild type and mutant transcripts in patient fibroblasts (lane p) and control cell cultures (lanes pc1 and pc2) were determined by RT-PCR. The wild type RT-PCR product is indicated by an arrow, mutant RT-PCR products are indicated by arrowheads. Sequencing of the aberrant transcripts showed retention of intron 2 (generating a 1.2 kb RT-PCR product) or activation of a cryptic splice site in exon 2 (generating a 288 bp RT-PCR product). (C) Western blot analysis of ephrin-B1 expression in patient fibroblasts and control cell culture lysates showed an approximately 50 kDa protein (indicated by an arrow). No smaller truncated protein was detected in patient fibroblasts. Protein sizes were determined using Precision Plus Protein™ Standards Dual Color (BIO-RAD). (D) The sequence of exon 2 and 3 and part of intron 2 are shown. Coding sequences are shown in capital letters, flanking sequences of intron 2 are shown in small letters. The *BfuA*I site at the mutation site is underlined. Aberrant splicing is indicated by green letters, the cryptic splice junction is underlined. Premature termination codons (STOP) generated by aberrant splicing or retention of intron 2 are highlighted in red.

Previously, it has been shown that PTC-causing mutations occurring in internal exons of *EFNB1 *cause transcript depletion by nonsense-mediated mRNA decay (NMD). Escape from NMD, however, has been observed for the c.196C > T/p.R66X mutation that is located in exon 2. In patient fibroblast cultures, a mutant *EFNB1 *transcript was detected by RT-PCR in addition to the wild type transcript (Figure 2A and 2B). Mutation c.614_615delCT is located in exon 4 of *EFNB1 *and also showed escape from NMD [25] (Figure 2C and 2D). In another girl from unaffected parents, *de novo *occurrence of the heterozygous splice-site mutation c.406+2T > C was detected in her genomic DNA (Figure 3A). Splice-site mutation c.406+2T > C alters the consensus splice donor site "GT" at the junction of exons 2 and 3. Analysis of the patient fibroblasts by RT-PCR revealed the wild type *EFNB1 *transcript to be the main transcript, but additional transcripts were derived from the mutant allele c.406+2T > C (Figure 3B). Cloning and sequencing demonstrated retention of intron 2, or activation of a cryptic splice site within exon 2, resulting in premature termination of ephrin-B1 (Figure 3D). Retention of intron 2 generated an 1.2 kb RT-PCR product. The same product has been shown previously for splice site mutation c.407-2A > T [25], that has been used as a control (pc1). As a second control (pc2) wt *EFNB1 *expressing fibroblasts were used. The band above 1.2 kb observed in all three samples was identified as a genomic DNA contamination.

Frameshift mutation c.614_615delCT and splice site mutation c.406+2T > C, both, were predicted to result in protein truncation preceding the transmembrane domain of ephrin-B1. This prompted us to investigate whether the observed transcripts will give rise to a truncated soluble ephrin-B1 protein product that may exhibit dominant-negative or gain-of-functions effects. To determine whether mutant transcripts give rise to a truncated ephrin-B1 protein, Western blot analysis was performed. In contrast to presence of wild type and mutant RNA in patient fibroblasts, only wild type but not a mutant truncated ephrin-B1 protein was detected for both mutations using a polyclonal anti-human ephrin-B1 antibody (Figure 2E, 3C). In addition to the 50 kDa protein, a smaller ≈40 kDa band was detected (Figure 3C). A faint band is also detected in Figure 2E and also can be seen in the control wt fibroblasts (Figure 3C, left lane). Presumably, this is an unglycosylated or degraded form of the wild type ephrin-B1. Taken together, Western blot results suggest that truncated ephrin-B1 is rapidly degraded in the patient fibroblasts. Absence of the mutant protein shows that mutations c.614_615delCT and c.406+2C > T appear to have a loss-of-function effect.

### Analysis of missense mutations in a cell culture model

**Figure 4:**
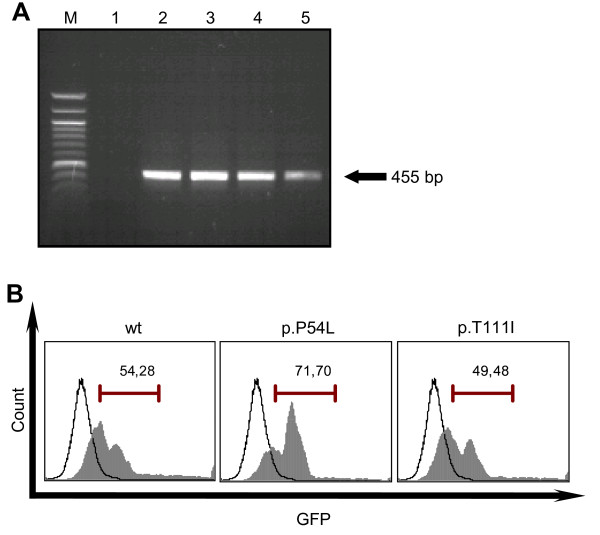
**Transfection of NIH 3T3 cells with the p.P54L and p.T111I *EFNB1 *cDNA constructs**. (A) *EFNB1 *constructs p.P54L and p.T111I were generated by site-directed mutagenesis and transfected into the NIH 3T3 cells. After the transfection, RT-PCR using *EFNB1 *specific primers was performed. RT-PCR products of the primary NIH 3T3 cells (lane 1), NIH 3T3 cells transfected with wild type, p.P54L, p.T111I *EFNB1 *cDNA constructs, respectively (lanes 2-4) and Cos-1 cells as a positive control (lane 5) are shown. Size markers are shown in lane M (100 bp DNA ladder, Invitrogen). (B) FACS analysis of the NIH 3T3 cells transfected with wild type and mutant *EFNB1 *constructs. Grey peaks show maximum of GFP fluorescence in NIH 3T3 transfected cells. Empty peaks show maximum of GFP fluorescence in untransfected control cells.

To determine the role of p.P54L and p.T111I missense mutations in EphB2/ephrin-B1 signalling, a cell culture model was established. Expression constructs containing wild type, p.P54L and pT111I *EFNB1 *cDNA, respectively, were generated by site-directed mutagenesis and used for transfection of NIH 3T3 cells. NIH 3T3 were chosen because they do not express mouse homologues of B-type ephrin genes (Figure 4A, [35]). Transfection efficiency of the constructs in NIH3T3 was monitored by RT-PCR and FACS analysis (Figure 4).

**Figure 5:**
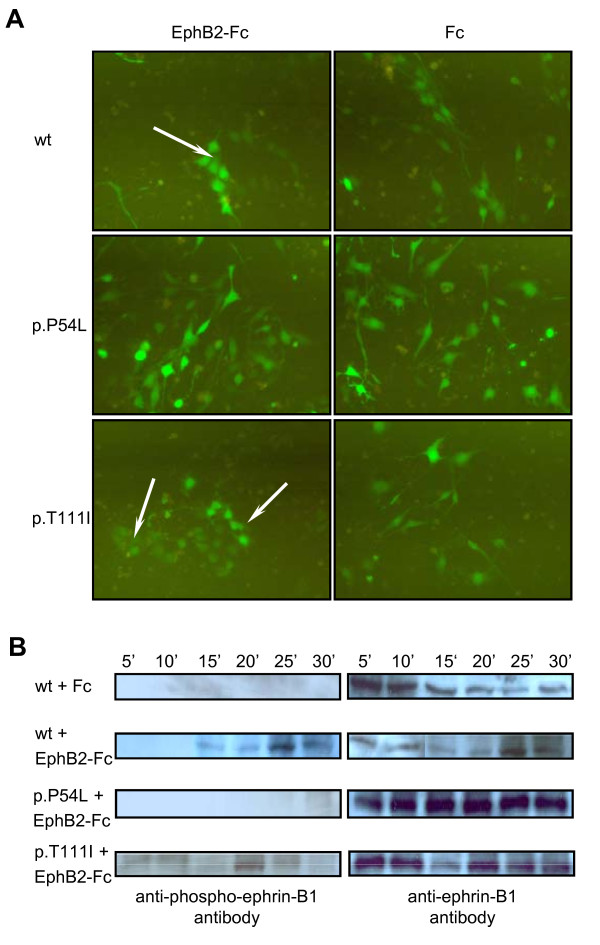
**EphB2-Fc stimulation of wild type, p.P54L, p.T111I ephrin-B1 expressing NIH 3T3 cells**. (A) NIH 3T3 cells expressing wild type and p.T111I ephrin-B1 were detected by fluorescent microscopy as cell clusters after the EphB2-Fc stimulation (indicated by arrows). Cells expressing p.P54L were scattered like the cells in the control (Fc treatment). (B) Following a time course of 5 to 30 min of EphB2-Fc stimulation, Western blot analysis of wild type, p.P54L and p.T111I ephrin-B1 expressing NIH 3T3 cells using phospho-ephrin-B (Tyr324/329) antibody and anti-ephrin-B1 antibody was performed. Cells transfected with wild type ephrin-B1 and treated with Fc only were used as a control. The anti-ephrin-B1 antibody (A-20) was used to demonstrate the whole amount of the ephrin-B1 protein loaded. Differences in the amount of ephrin-B1 on the Western blots can be explained by unequal amounts of ephrin-B1 in the lysates. This may be due to differences in the expression from the transiently transfected *EFNB1 *cDNA plasmids. Protein sizes were determined using Precision Plus Protein™ Standards Dual Color (BIO-RAD).

To understand the impact of missense mutations on cell behaviour and ephrin-B1 reverse signalling, wild type, p.P54L and p.T111I ephrin-B1 expressing NIH3T3 cells were stimulated with EphB2-Fc. Wild type and p.T111I ephrin-B1 expressing NIH3T3 cells were forming clusters (Figure 5A), whereas cells expressing p.P54L ephrin-B1 were scattered much like the ephrin-B1 expressing cells in the control following Fc-only treatment. To determine the impact of p.P54L and p.T111I missense mutations on Tyr-phosphorylation, wild type, p.P54L and p.T111I *EFNB1 *expressing NIH 3T3 cells were stimulated with pre-clustered EphB2-Fc from 5 to 30 min. After the stimulation, Tyr324 and Tyr329 phosphorylation response was monitored by Western blot analysis using Tyr324/329-specific polyclonal antibodies (Figure 5B). This showed phosphorylation of the wild type ephrin-B1 to peak at about 25 min after stimulation. We observe reduced phosphorylation in p.T111I lysates compared with wild type lysates at 30 min despite about equal amounts of ephrin-B1 protein detected by anti-ephrin-B1 antibody (Figure 5B second row of upper panel). In p.T111I expressing cells the level of Tyr-phosphorylation appears lower than in wild type *EFNB1*-transfected NIH 3T3 and Tyr-phosphorylation seems to last for a shorter period of time, e.g. in p.T111I cells only a weak signal was detected at time point 30 min in contrast to the wild type cells. For p.P54L mutant ephrin-B1 no phosphorylation was detected like in the controls following Fc-only treatment.

## Discussion

In this study, we examined the impact of disease-causing mutations in the ephrin-B1 gene. The major types of mutations including frameshift, nonsense and splice site mutations generate PTCs that elicit NMD. Usually, NMD proceeds when the PTC occurs in internal exons or is located more than 50-55 bp upstream the exon-intron junction of the penultimate exon [36]. Unexpectedly, in this work it was shown that the c.196C > T/p.R66X mutation escapes NMD. This mutation was described before in both familial and sporadic cases [3,20,22,23]. A PTC that is caused by c.196C > T/p.R66X is located in the second exon 208 bp upstream the exon-intron junction. The NMD escape of the PTC-causing mutations that are located in the second exon appears to be not unique for the *EFNB1 *gene. Kang and Macdonald described such mutations in *GABRA1 *and *GABRG2 *genes [37], and Jensen et al. in the *JARID1C *gene [38]. The reason for NMD escape is still not clear. Bühler et al. [39] proposed a NMD-promoting element (NPE) in exon 2 of the *IGHM *gene. PTCs located downstream of the NPE will elicit NMD, whereas PTCs located upstream of the NPE will result in NMD escape. This has been experimentally proven by deletion analysis demonstrating NMD failure upon removal of the NPE from exon 2. We may propose that a NPE also exists in *GABRA1*, *GABRG2*, *JARID1C *and *EFNB1 *and that PTCs that are located upstream of this element do not elicit NMD.

Another possible explanation for the NMD escape was raised by Zhang and Maquat [40]. This group showed that NMD in the *TPI *gene can be avoided by the re-initiation of the translation by the 14^th ^AUG codon including the Kozak sequence. In *EFNB1*, translation re-initiation could occur at the 156^th ^codon (AUG). This could lead to the synthesis of a truncated ephrin-B1 that lacks the signal peptide and almost the entire extracellular domain. However, such a protein will not enter the endoplasmatic reticulum and consequently will not appear on the cell surface.

Escape from NMD also could give rise to truncated, soluble ephrin-B1 polypeptides that lack the transmembrane and intracellular domain. Such polypeptides could exhibit dominant-negative or gain-of function effects. Frameshift mutation c.614_615delCT occurred in exon 4 of *EFNB1 *and generates a PTC but the transcripts escape NMD. Expression analysis of patient fibroblasts revealed transcripts from mutant and wild type alleles at similar amounts, whereas only the wild type but no truncated ephrin-B1 was detected by Western blot analysis. This rather suggests that truncated ephrin-B1 proteins are unstable and do not contribute substantially to the CFNS phenotype. In another female patient with classical CFNS phenotype, splice site mutation c.406+2T > C was detected. In the patient examined here c.406+2T > C occurred *de novo*. Expression analysis of the patient's fibroblasts revealed retention of intron 2 as has been previously detected for the splice acceptor "AG" mutation c.407-2A > T at the same exon junction [25]. In addition, activation of a cryptic splice site in the preceding exon 2 was detected for c.406+2T > C, which is generally a frequent consequence of 5' splice site mutations [41,42]. Cryptic splice site activation has not been observed for wt *EFNB1*, in fact, only a single *EFNB1 *transcript has been reported [30]. Both, intron retention and cryptic splice site activation resulted in PTCs and reduced transcript amounts when compared with the wild type allele.

Like c.614_615delCT no truncated soluble ephrin-B1 polypeptide was generated from c.406+2T > C *EFNB1 *mutation in patient fibroblasts. Mutation c.196C > T/p.R66X generates a PTC just 36 amino acids following the signal peptide. Presumably, this will not allow production of a functional polypeptide, however, we were not able to further analyse it because the polyclonal anti-ephrin-B1 antibody we used does not recognize the N-terminal part of ephrin-B1.

The impact of two missense mutations on ephrin-B1 signalling and cell behaviour was studied in a cell culture model using NIH 3T3 fibroblasts. Reverse signalling influences actin cytoskeletal rearrangement and may result in transcriptional regulation of different genes involved in extracellular matrix reorganization [12,43]. We performed Western blot analysis to analyse phosphorylation of Tyr324 and Tyr329 of wild type, p.P45L and p.T111I proteins in response to EphB2-Fc receptor stimulation. No Tyr324/329 phosphorylation of p.P54L mutant protein was detected. In contrast, p.T111I ephrin-B1 still showed Tyr324/329 phosphorylation like the wild type protein, albeit the timing appeared slightly different. Since p.T111I undoubtedly causes the CFNS phenotype, phosphorylation of Tyr324/329 may be less important for disease manifestation.

Altogether, eight different mutations were functionally analysed at the mRNA and protein level, respectively (Table 1). Most of them appear to result in loss of gene function, but additional mechanisms are involved in manifestation of CFNS. There is some evidence that CFNS develops as a consequence of cellular interference, hence the missense mutations were further investigated in cell culture.

**Table 1:** Summary of the functionally analysed *EFNB1 *mutations

Mutation	Exon/intron	Cell type	*EFNB1 *mRNA	Ephrin-B1 protein	References
c.196C > T p.R66X	Exon 2	Patient fibroblasts	wt level	N.d.	This report
c.377_384 delTCAAGAAG	Exon 2	Patient fibroblasts	Strongly reduced amount (NMD)	N.d.	[21]
c.614_615 delCT	Exon 4	Patient fibroblasts	wt level (NMD escape)	No protein detected	This report, [21]
c.406+2T > C	Intron 3	Patient fibroblasts	Reduced amount	No protein detected	This report
c.407-2A > T	Intron 3	Patient fibroblasts	Strongly reduced amount (NMD)	N.d.	[21]
c.161C > T p.P54L	Exon 2	Patient fibroblasts and transfected NIH 3T3 cells	wt level	Protein, but no EphB2-activated phosphorylation	This report, [21]
c.332C > T p.T111I	Exon 2	Transfected NIH 3T3 cells	N.d.^a^	Protein, but altered EphB2-activated phosphorylation	This report
c.409A > G p.T137A	Exon 3	Patient fibroblasts	wt level	N.d.	[21]

Not determined; ^a^Not determined in patient fibroblasts.

NIH3T3 cells expressing either wild type or mutant ephrin-B1 exhibited differences in cluster formation after EphB2-Fc stimulation. Cells expressing wild type or mutant ephrin-B1 were visualized by the green fluorescent protein and showed a scattered distribution in tissue culture dishes before stimulation. EphB2-Fc stimulation induced formation of clusters in the wild type and p.T111I protein expressing cells, whereas no cell clusters were found in p.P54L expressing cells. This suggests that phosphorylation of Tyr324/329 is closely linked with the cluster formation, however, the mechanism for this is unclear. Possible reasons are that wild type and p.T111I expressing cells experience a proliferative signal upon EphB2 stimulation. After division daughter cells do not move apart but rather stay close together. Alternatively, cells respond to EphB2 stimulation with increased motility and migrate into clusters. Embryonic mouse cells expressing an ephrin-B1 lacking the most C-terminal PDZ binding domain do not sort-out from wild type cells, whereas ephrin-B1 null cells do [44,45,9]. In this respect, it is striking that missense mutations detected in CFNS patients have been detected exclusively in the exons encoding the extracellular region of ephrin-B1, which strongly argues for the involvement of Eph receptor forward signalling in the pathogenic mechanism. We propose that CFNS is caused mostly by disturbance of Eph receptor forward signalling and the consequences of cellular interference in heterozygous females.

## Conclusions

In this work three PTC-causing mutations were analysed: nonsense c.196C > T/p.R66X, frameshift c.614_615delCT and splice-site c.406+2T > C mutation. These mutations give rise to the mutant RNA, but no mutant protein was detected. According to these results and previously published data, it can be concluded, that the majority of PTC-causing *EFNB1 *mutations have neither dominant-negative, nor gain-of-function effects but rather loss-of-function effect.

The analysis of missense mutations p.P54L and p.T111I revealed that both cause CFNS but have different mechanisms of ephrin-B1 disturbance of signalling. Mutation p.P54L seems to have loss-of-function effect since no Tyr324/329 phosphorylation of the p.P54L ephrin-B1 and no cluster formation of the p.P54L expressing cells were shown, whereas p.T111I ephrin-B1 differs slightly from the wild type in phosphorylation timing. Therefore, additional mechanisms involved in phenotypic manifestation need to be postulated. This may include other tyrosine residues of ephrin-B1 are more important for reverse signalling. Another possibility could be impaired forward signalling of Eph receptor expressing cells. Combined with cellular interference this may be the main pathogenic mechanism in CFNS manifestation in female patients.

## Competing interests

The authors declare that they have no competing interests.

## Authors' contributions

RM and IW designed and performed the experiments; patients material and information was provided by BS, ZG, VT and PW. The manuscript was written by RM and IW and read and approved by all authors.

## Pre-publication history

The pre-publication history for this paper can be accessed here:

http://www.biomedcentral.com/1471-2350/11/98/prepub
